# A Low-G Silicon Inertial Micro-Switch with Enhanced Contact Effect Using Squeeze-Film Damping

**DOI:** 10.3390/s17020387

**Published:** 2017-02-16

**Authors:** Yingchun Peng, Zhiyu Wen, Dongling Li, Zhengguo Shang

**Affiliations:** 1 Microsystem Research Center, Chongqing University, Chongqing 400044, China; wzy@cqu.edu.cn (Z.W.); lidongling@cqu.edu.cn (D.L.); zhengry@cqu.edu.cn (Z.S.); 2 Key Laboratory of Fundamental Science of Micro/Nano-Device and System Technology, Chongqing University, Chongqing 400044, China

**Keywords:** inertial switch, acceleration switch, MEMS, contact time, squeeze-film damping

## Abstract

Contact time is one of the most important properties for inertial micro-switches. However, it is usually less than 20 μs for the switch with rigid electrode, which is difficult for the external circuit to recognize. This issue is traditionally addressed by designing the switch with a keep-close function or flexible electrode. However, the switch with keep-close function requires an additional operation to re-open itself, causing inconvenience for some applications wherein repeated monitoring is needed. The switch with a flexible electrode is usually fabricated by electroplating technology, and it is difficult to realize low-g switches (<50 g) due to inherent fabrication errors. This paper reports a contact enhancement using squeeze-film damping effect for low-g switches. A vertically driven switch with large proof mass and flexible springs was designed based on silicon micromachining, in order to achieve a damping ratio of 2 and a threshold value of 10 g. The proposed contact enhancement was investigated by theoretical and experimental studies. The results show that the damping effect can not only prolong the contact time for the dynamic acceleration load, but also reduce the contact bounce for the quasi-static acceleration load. The contact time under dynamic and quasi-static loads was 40 μs and 570 μs, respectively.

## 1. Introduction

MEMS (micro-electro-mechanical system) technology-based inertial switches have great potential for acceleration sensing applications [1,2] due to its miniaturization, high integration level, and low or even no power consumption [3,4]. The inertial micro-switch typically consists of a proof mass suspended by springs as the movable electrode. When an acceleration exceeding a certain level (threshold value) is applied along the sensitive direction, the proof mass moves toward the substrate, and collapses onto the fixed electrode on the substrate, thus turning the switch on and establishing an electrical path in the external circuit. The contact time between the two electrodes is one of the most important properties for the switch, which should be long enough such that it is recognized by the external circuit. Since the first inertial micro-switch was reported in 1972 [5], a great number of inertial micro-switches have been developed, and they can be simply grouped into two categories: persistent switches, wherein the switch was designed with a keep-close function that can keep it closed after the acceleration event is over, and intermittent switches, wherein the switch re-opens after the acceleration dissipates. Persistent switches such as the latching switch [6,7,8], the bi-stable switch [9,10], and the micro-fluidic switch [11,12] has an excellent contact effect but usually requires an additional operation or structure to re-open itself, resulting in inconvenience for some applications wherein repeated monitoring is needed. On the other hand, the intermittent switch can sense the acceleration theoretically for an unspecified number of iterations. However, its moveable electrode bounces back after impacting the fixed electrode (the phenomenon of contact bounce), resulting in short contact time. Particularly, the contact time of the traditional intermittent switch with a rigid electrode is usually less than 20 μs due to the rigid contact process [13,14]. Moreover, the contact time is affected by the duration of the input load. Generally, the contact time is short when the switch is activated by dynamic acceleration load (short acceleration duration), while the contact bounce becomes the main problem in the case of quasi-static acceleration load (long acceleration duration) [15]. The contact bounce may also damage the interface of the two electrodes by mechanical hammering and electrical arcing, subsequently affecting the durability of the system, and possibly leading to permanent adhesion between the two electrodes [16].

In recent years, in order to enhance the contact effect of the intermittent switch, there has been considerable effort put into the inertial micro-switch with a flexible electrode fabricated by the multi-layer process of electroplating technology [14,17,18,19]. The contact effect can be improved by the deformation of the flexible electrode during the contact process. However, these switches usually focus on the threshold value above 100 g, while seldom aiming to sense an acceleration below 50 g (also named as low-g switch in this paper), which is required for such applications as geriatric healthcare [1] and automotive airbags [2]. According to the static equilibrium equation *a_th_* = *kx*_0_/*m* (where *a_th_* is the threshold acceleration, *k* is the spring constant, *x*_0_ is the distance between the two electrodes, and *m* is the mass of the proof mass), a low-g switch requires flexible springs and large proof mass. Such structure feature is hard to achieve with electroplating technology because the large proof mass requires a great number of electroplating processes, which can result in unexpected fabrication errors in the device, such as structure deformation induced by residual stresses between each electroplating layer.

In this paper, we aim to improve the contact effect of the inertial micro-switch for low-g acceleration sensing using a squeeze-film damping effect. The objective is to prolong the contact time for dynamic acceleration load and reduce the contact bounce for quasi-static acceleration load. A vertically driven switch with a large proof mass suspended by four serpentine springs was designed based on silicon micromachining, in order to achieve a damping ratio of 2 and a threshold value of 10 g. The dynamics of the switch was investigated by the finite-element-method (FEM) simulation and measured by a shacking system. The experimental results show that the contact time under the dynamic and quasi-static loads was 40 μs and 570 μs, respectively. The study in this paper can provide guidance in the design of inertial micro-switches where low-g sensing and long contact time are required.

## 2. Design and FEM Simulation

### 2.1. Device Design

**Figure 1:**
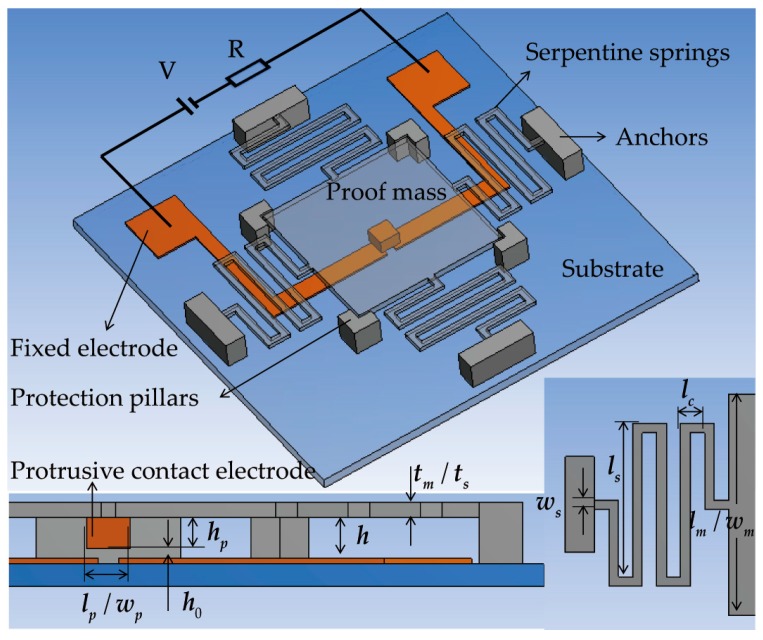
3-D sketch and main geometric specifications of the micro-switch.

The switch is designed to be vertically driven to employ the squeeze-film damping effect (i.e., a proof mass suspended by springs as the sensing element moving toward the substrate to sense out-of-plane acceleration), since the slid-film damping effect involved by laterally driven switches is so weak that it is usually neglected. The movement of the proof mass in the horizontal insensitive directions is limited by four fixed pillars. The size of the proof mass was 2300 μm in length and width, and 20 μm thick, which is large enough for the low-g sensing. Four serpentine springs 20 μm thick, which have a much lower spring constant than the typical cantilever beam, were used to support the proof mass from the anchors. The thickness of the proof mass is identical to that of the springs for facilitating fabrication. This structure feature can easily realize the threshold value of 10 g, which is the target threshold in this paper. A protrusion positioned at the bottom center of the proof mass serves as the moveable contact electrode. Two separated metal strips on the glass substrate form the double-contact-configuration fixed electrode. An acceleration greater than the threshold value can cause the proof mass to move toward the substrate, traversing the gap between the two electrodes, making the moveable electrode short the fixed electrode, thus turning the switch on. A sketch and the main geometric specifications of the designed switch are shown in Figure 1. In the sketch, the proof mass and springs are set as transparent structures to display the two electrodes under them.

The dynamic threshold acceleration in Equations (1) provides the essential information about the dynamic behavior of the inertial switch [20]:

(1) $$a_{th}=\sqrt{{\left(\omega_{n}{}^{2}-\omega^{2}\right)}^{2}+{\left(c\omega /m\right)}^{2}}\cdot h_{0}$$

where $\omega_{n}=\sqrt{k/m}$ is the natural angular frequency of the switch, *k* is the spring constant, *m* is the mass of the proof mass, ω is the angular frequency of the applied acceleration, and *c* is the squeeze-film damping viscous coefficient. It can be observed that the electrode-gap height (*h*_0_) is an additional design parameter for setting the threshold level under certain acceleration frequency. In the device design, the electrode-gap height is adjusted by modifying the protrusion height according to the relationship of *h*_0_ = *h* − *h_p_* (Figure 1), when the air-gap height (*h*) is fixed for designing the required damping effect (as explained in the following paragraphs). By ignoring the mass of the protrusion (due to its small size), the changing of the protrusion height has no effect on the natural frequency of the switch.

The squeeze-film damping effect is caused by the air surrounding the structure when the proof mass moves toward the substrate. The flow mechanism of the air is usually measured by the Knudsen number, *K_n_* = λ/*d*, where λ ≈ 0.1 μm is the mean free path of the air under 1 atm (standard atmospheric pressure) [21], and *d* is the initial air-gap height. As seen in Figure 1, two fluids should be considered: the fluid between the proof mass and substrate, and the fluid between the protrusion and substrate.

In the device structure, the air-gap height (*h*) is at least 35 μm. As a result, we have *K_n_* < 0.01, and the fluid between the proof mass and substrate can be considered as a continuum [22]. The behavior of the continuous fluid can be governed by the linearized Reynolds equation, based on the hypotheses of small amplitude displacement of the proof mass and small squeeze number [23], which is applicable for this study. Therefore, according to the linearized Reynolds equation, the expression of the squeeze-film damping viscous coefficient for the oscillating proof mass is [23]:

(2) $$c=\frac{\mu l_{m}w_{m}{}^{3}}{h^{3}}\eta \left(w_{m}/l_{m}\right)$$

where $\mu =1.81\;\times \;10^{-5}\;\mathrm{Pa}\cdot s$ is the viscosity of the air under 1 atm and 20 °C [21], and $\eta \left(w_{m}/l_{m}\right)$ is a correction factor and is equal to 0.42 when $w_{m}=l_{m}$. Note that c is inversely proportional to the third power of h. As such, the air-gap height (h) is a strong design parameter to set the value of the squeeze-film damping ratio ζ, which is defined as $\zeta =c/2\omega_{n}m$.

In contrast, the electrode-gap height (*h*_0_) is only several micrometers. Thus, according to the definition of the Knudsen number, the fluid between the protrusion and substrate was regarded as a slip flow, and the fluid rarefaction effect [24] should be taken into account. Furthermore, the displacement of the protrusion is equal to the initial electrode-gap height (in order to contact the fixed electrode and thus turn the switch on), and the large amplitude displacement effect [25] should also be considered. By substituting the standard fluid viscosity with an effective term [26] and increasing the damping viscous coefficient by a factor [25], it was found that the corresponding damping ratio is on the order of 0.001. As such, the damping effect between the protrusion and substrate is neglected.

From the above analysis, we have the design strategy of the switch as follows: first, the springs and proof mass are designed to achieve a low natural frequency of the switch in order to meet the low-g requirement; second, the air-gap height (*h*) between the proof mass and substrate is designed for the required damping effect to improve the contact effect (as will be seen in the next subsection); third, the electrode-gap height (*h*_0_) is designed to achieve the objective threshold acceleration under certain acceleration frequency, when the natural frequency and damping effect of the switch are fixed. These structure features facilitate the single factor comparison of different dynamic responses of the switch under various damping effects and acceleration loads.

### 2.2. FEM Simulation

**Figure 2:**
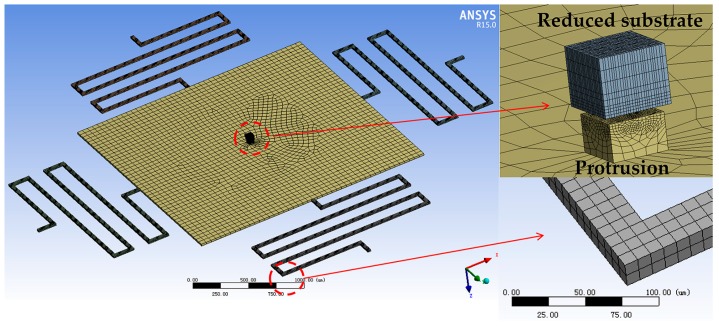
Finite element model of the micro-switch with the contact pair of the protrusion and reduced substrate.

The switch was modeled by the software of ANSYS Workbench, as shown in Figure 2. The end sections of the four suspended springs and the backside of the substrate were constrained to be zero in all degrees of freedom. The protrusion and the substrate were defined as the contact pair, and the contact type was frictionless-solid. Transient analysis was introduced to investigate the dynamics of the switch. Due to the deformation (even slight) in the interface of the contact pair during the contact process, the simulation can be time-consuming. As such, in order to guarantee the computational accuracy and save the computational cost, the area of the substrate was reduced such that it was only slightly larger than that of the protrusion. More importantly, the device structure was divided into six parts (4 parts of the four springs, 1 part of the proof mass and protrusion, and 1 part of the substrate) by the method of creating slice, and the element size of each part was separately defined: by the method of body sizing, the element size of the four springs was set at 10 μm, and the element size of the proof mass and protrusion was set at 50 μm; by the method of face sizing, the element size of the four side faces of the protrusion was set at 10 μm; by the method of contact sizing, the element size of the contact pair was set at 2 μm. After the element-size definition, the model was meshed by the method of Hex Dominant.

For the transient analysis, the time integration method of iterative algorithm (solver type) was used to improve the stability and accuracy of the results. The damping effect was introduced by the so called Rayleigh damping coefficients—alpha damping coefficient $\alpha =2\zeta \omega_{n1}\omega_{n2}/(\omega_{n1}+\omega_{n2})$ and beta damping coefficient $\beta =2\zeta /(\omega_{n1}+\omega_{n2})$, where $\omega_{n1}$ and $\omega_{n2}$ are the first and second order natural angular frequencies of the switch, respectively [27]. The alpha and beta coefficients correspond to the mass and stiffness coefficients, respectively, in Workbench. The load apply step was also optimized to guarantee computational accuracy and save computational cost: first, the total step number of the acceleration load was set at 30; second, the numbers of the substep (including initial substep, minimum substep, and maximum substep) were set by two schemes—2, 1, and 3, respectively, for the case when the contact pair were separated from each other, and 10, 6, and 12, respectively, for the case when the contact pair were in contact with each other; third, the step end time of the analysis settings was set according to the acceleration duration. By applying different mass and stiffness coefficients, and acceleration loads of various amplitudes and durations, the displacement responses (including contact behavior) of the switch under different damping ratios and acceleration loads (including dynamic and quasi-static loads) were obtained. Considering that the first order frequency of the switch is 808 Hz, 1 ms and 5 ms acceleration durations were chosen to represent the dynamic and quasi-static loads, respectively.

The main geometric parameters and material properties of the designed switch used in this simulation are shown in Table 1 and Table 2, respectively. The variables of *h* and *h*_0_ were determined to meet the required damping ratio and threshold value, respectively.

**Figure 3:**
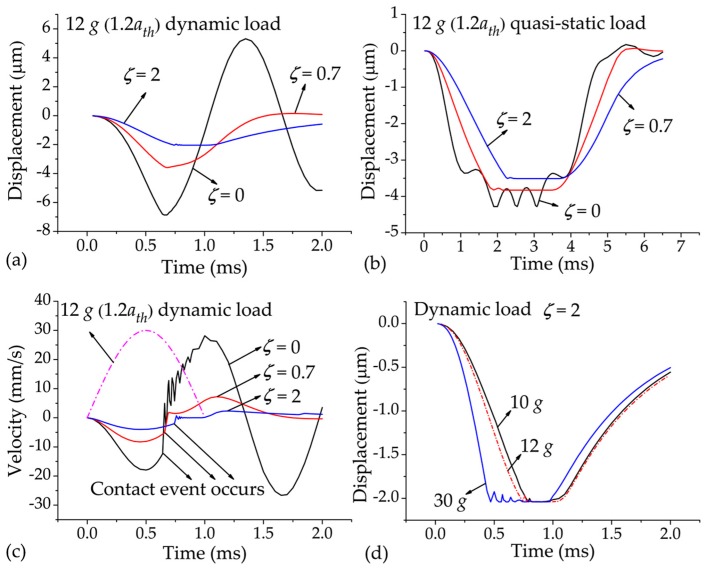
(**a**,**b**) Displacement responses of the switch (ζ = 0, 0.7, and 2) under the accelerations of (**a**) 12 g (1.2 *a_th_*) dynamic load and (**b**) 12 g (1.2 *a_th_*) quasi-static load; (**c**) Velocity responses of the switch (ζ = 0, 0.7, and 2) under 12 g (1.2 *a_th_*) dynamic load; (**d**) Displacement responses of the switch (ζ = 2) under 10 g, 12 g, and 30 g dynamic loads.

Figure 3a,b show the displacement responses of the switch to the dynamic and quasi-static loads, respectively, for the cases when ζ = 0 (*h* = ∞), 0.7 (*h* = 49.2 μm), and 2 (*h* = 35 μm). ζ = 0 is the case where the squeeze-film damping effect is neglected, ζ = 0.7 is the most representative value employed by many researchers in this field, and ζ = 2 is used to provide a great enough damping effect. In Figure 3a,b, it was found that the movement of the protrusion was stopped by the substrate, and a period of contact process then occurred. The applied acceleration was 12 g, which is 20% overload of the threshold value (*a_th_*) in order to make sufficient contact behavior. It can be seen that the contact time is prolonged with increasing damping ratio when the switch is excited by the dynamic load (Figure 3a). The contact bounce is reduced with increasing damping ratio, and the contact time is prolonged in the case of quasi-static load (Figure 3b). When ζ = 2, the contact time under the dynamic and quasi-static loads were about 210 μs and 1300 μs, respectively.

The role of the squeeze-film damping effect can be revealed by the velocity response of the protrusion to the dynamic load. Figure 3c indicates that the velocity is decreased with increasing damping ratio; the velocity when ζ = 2 is much smaller than those when ζ = 0 and 0.7 and, as such, can be further decreased by the continuous action of the acceleration after the contact event occurs, subsequently keeping zero in a period of time. The duration of the zero velocity, of course, corresponds to the contact time between the protrusion and substrate. However, when the applied acceleration is much higher than the threshold value, the velocity of the protrusion will be significantly increased. At this point, contact bounce occurs, thus reducing the contact effect as shown in Figure 3d.

Note from Figure 3a,b that for the expected threshold value of 10 *g*, the required electrode-gap height changes with the damping ratio and the input load duration, which is in accord with the analysis of Equation (1). In the experimental part, the switch of *h*_0_ = 2 μm and *h* = 35 μm was fabricated and tested. Simulation results indicate that the threshold and contact time of this switch was 10 g and 150 μs under the dynamic load, and 5.8 g and 860 μs under the quasi-static load.

## 3. Fabrication

**Figure 4:**
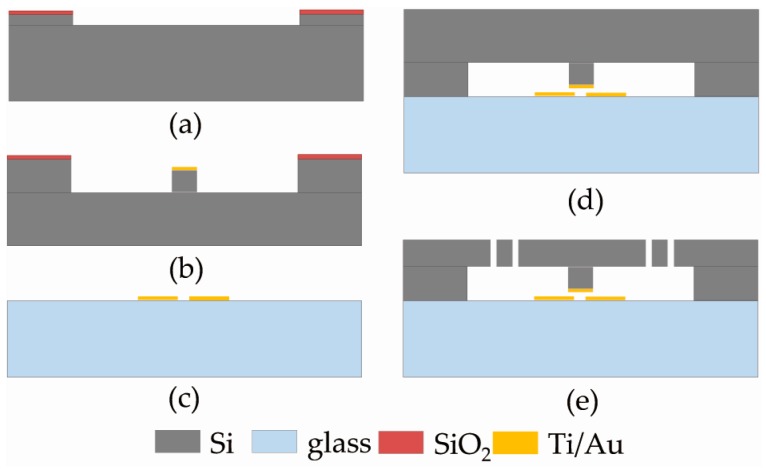
Process sequence for the fabrication of the micro-switch.

**Figure 5:**
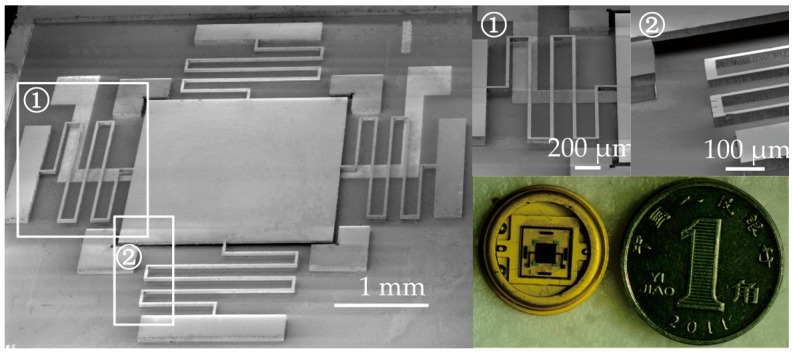
SEM and optical photographs of the fabricated and packaged micro-switches.

The switch was fabricated using a typical silicon-on-glass process. The main fabrication processes are shown in Figure 4 and are described as follows. (a) A 300 nm thick silicon-oxide layer was deposited and patterned on a silicon wafer (4-inch diameter, 500 μm thick, <100> orientation) by thermal oxidation and buffered HF solution, respectively. By using the silicon-oxide layer that served as a mask, a 2 μm deep recess was carried out by wet-etching (TMAH) to create the electrode gap. (b) A titanium/gold layer (40/120 nm thick) was sputtered and patterned by lift-off technology, forming the metal film of the protrusive contact electrode. Subsequently, a 33 μm height protrusion was constructed by an inductively coupled plasma (ICP) etch. Thus, the 35 μm air gap between the proof mass and substrate was created. (c) The fixed electrode on a glass wafer (500 μm thick) was realized by sputtering and patterning a titanium/gold layer (40/120 nm thick) using lift-off technology. (d) The silicon-oxide layer on the silicon wafer was removed by a buffered HF solution, and the two processed wafers (silicon and glass) were anodically bonded at 350 °C, 10^−2^ mbar and −850 V. A peripheral frame (outside the main device structure and contained within the bonding region) was used to protect the front side of the silicon wafer during the post processes by establishing a seal chamber between the two wafers. (e) The silicon wafer was thinned in KOH solution to determine the thickness of the springs and proof mass (20 μm). Then, by using a 3 μm thick positive photoresist as a mask, the device was completely released by ICP etch. After the photoresist was removed, the switch was packaged by a transistor-outline enclosure with six client leads for wire bonding. Figure 5 shows the fabricated and packaged micro-switches.

## 4. Results and Discussion

**Figure 6:**
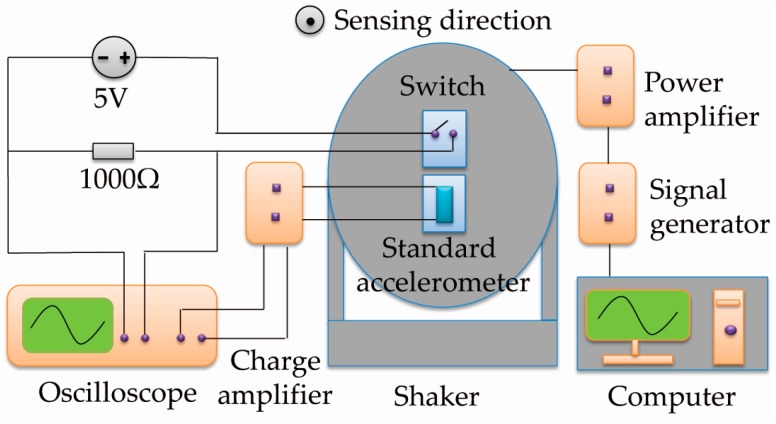
Schematic diagram of the test setup.

The fabricated switch was tested by a shaking system. A schematic diagram of the test setup is shown in Figure 6. The switch in a device holder was mounted on the shaking table, and electrically connected to a DC power supply (5 V) and a sensing resistor (1000 Ω). A signal generator was used to offer a half-sine wave to simulate the acceleration signal. The acceleration was first amplified by a power amplifier, and then applied to the shaker to produce the required vibration. The vibration was detected by a standard accelerometer (100 mV/g) fixed on the shaking table beside the switch. The output of the accelerometer was amplified by a charge amplifier to facilitate the signal collection. The acceleration was controlled by a computer and can be set from 0.1 to 30 g with an accuracy of 0.1 g. In order to accurately obtain the threshold value of the switch, the acceleration was first set to a level below the threshold, and then gradually increased with increment of 0.1 g until the switch was observed to turn on (output: 5 V). The real-time data (both the switch and accelerometer outputs) was recorded by a multichannel oscilloscope, and each experimental datum was repeated at least 10 times.

**Figure 7:**
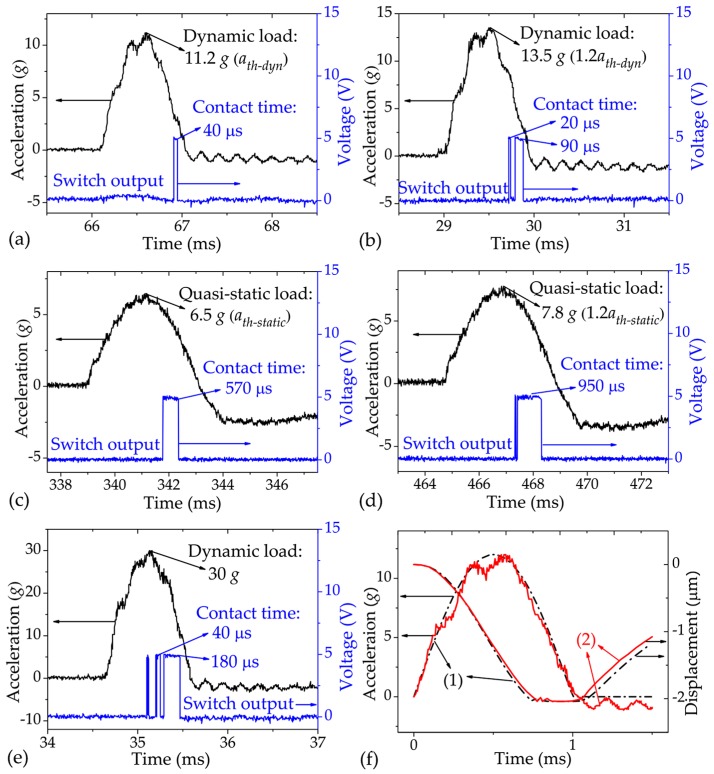
(**a**–**e**) Measured results of the fabricated switch under accelerations of (**a**,**b**) 11.2 g (*a_th−dyn_*) and 13.5 g (1.2 *a_th−dyn_*) dynamic loads; (**c**,**d**) 6.5 g (*a_th−static_*) and 7.8 g (1.2 *a_th−static_*) quasi-static loads; (**e**) 30 g dynamic load. (**f**) Simulation results of the displacement response of the switch under (1) the standard and (2) the practical acceleration loads of 12 g amplitude and 1 ms duration.

Figure 7a–d indicate the typical test results of the fabricated switches. For the case of dynamic load: the measured threshold and the corresponding contact time were 11.2 g (*a_th−dyn_*) and 40 μs, respectively, as shown in Figure 7a; the contact time was prolonged to 90 μs under the acceleration of 13.5 g (1.2 *a_th−dyn_*), as shown in Figure 7b. The measured contact time is much longer than that of the traditional design, in which the damping effect is neglected and the contact time is usually less than 20 μs [13,14]. For the case of quasi-static load, the contact bounce is considerably reduced compared with the traditional design [14], as shown in Figure 7c,d; the measured threshold and the corresponding contact time were 6.5 g (*a_th−static_*) and 570 μs, respectively, as shown in Figure 7c; the contact time was prolonged to 950 μs under the acceleration of 7.8 g (1.2 *a_th−static_*), as shown in Figure 7d. The test results indicate that the design objective can be realized by the contact-enhanced design in this paper.

Figure 7e shows the test result of the switch under a higher dynamic load of 30 g. The switch output has a more serious contact bounce than those shown in Figure 7a,b, in accordance with the simulation results shown in Figure 3d. As mentioned in Section 1, since the contact bounce may damage the interface of the two electrodes or even cause the switch to fail [16], acceleration load much higher than the threshold value applied to the switch should be avoided.

The measured contact time is shorter than the simulation results. To be specific, the measured contact time is shorter than half of the simulation results when the switch is activated by the dynamic load; however, the measured contact time is longer than half of the simulation results in the case of the quasi-static load. Note from Figure 7a–d that, compared with the dynamic load, the quasi-static load is much more similar to the standard half-sine wave, and there are some small bounces in the peak of the switch output. As such, we consider that the shorter measured contact time is probably caused by the un-standard wave of applied acceleration loads. In order to further investigate this issue, the dynamic response of the switch under the un-standard acceleration load (extracted from the oscilloscope) was simulated by the ANSYS Workbench. Figure 7f compares the obtained result (the solid line) with that of the switch under the standard half-sine wave (the dotted line). The following can be seen: first, the practical acceleration load is much rougher than the standard wave; second, the average amplitude of the practical acceleration load (7.33 g) is lower than that of the standard wave (7.75 g). As seen in Figure 7f, the two deviations of the practical acceleration load from the standard wave can cause a larger rebound amplitude (or additional small bounces) of the movable electrode, and shorten the contact process, resulting in a shorter contact time.

The measured thresholds under the dynamic and quasi-static loads were 11.2 g and 6.5 g, respectively, which are higher than the simulation results of 10 g and 5.8 g, respectively. The threshold discrepancy can be attributed to several factors. The first is the applied un-standard acceleration load, since its average amplitude is lower than that of the standard wave as mentioned above. The second is the variation of the 35 μm air-gap height due to the fabrication error of the 33 μm protrusion height. FEM simulation shows that variations as small as 0.5 μm in the air-gap height can cause a change of ca. 0.6 g in the threshold value under the same dynamic load. This issue is no surprise because the threshold value becomes sensitive to the variation in the air-gap height as the damping effect is significantly increased. The third is the residual stresses in the springs, which will make the spring stiffer and then increase threshold value. These issues will be further studied in the future. However, because the main concern in this paper is the prolongation of the switch, the experimental results can sufficiently support the contact-enhanced design.

## 5. Conclusions

In order to improve the contact effect of the inertial micro-switch for low-g acceleration sensing, a vertically driven silicon switch with a damping ratio of 2 and threshold value of 10 g was designed in this paper. The contact enhancement was investigated by the simulation of ANSYS Workbench. The simulation results show that the damping effect can prolong the contact time when the switch is excited by dynamic load, and reduce the contact bounce in the case of quasi-static load. The switch was fabricated by a typical silicon-on-glass process, and measured by a shaking system. The test results are in agreement with the simulation results. The measured threshold value and contact time were 11.2 g and 40 μs, respectively, under a 1 ms dynamic acceleration load, and 6.5 g and 570 μs, respectively, under a 5 ms quasi-static acceleration load. The contact time was considerably prolonged compared with the traditional switches in which the damping effect is neglected, and the contact time is usually less than 20 μs.

## Figures and Tables

**Table 1:** Main geometric parameters of the micro-switch (μm).

$l_{m}/w_{m}$	$l_{p}/w_{p}$	$t_{m}/t_{s}$	$l_{s}$	$l_{c}$	$w_{s}$	h	$h_{0}$	$h_{p}$
2300	50	20	1600	150	30	Variable	Variable	$h-h_{0}$

**Table 2:** Main material properties of the device structure.

Material	Density	Young’s Modulus	Poisson’s Ratio
Silicon	2330 kg/m^3^	169 GPa	0.28
Glass	2200 kg/m^3^	70 GPa	0.17
